# Assessment of gene–disease associations and recommendations for genetic testing for somatic variants in vascular anomalies by VASCERN-VASCA

**DOI:** 10.1186/s13023-024-03196-9

**Published:** 2024-05-22

**Authors:** Nicole Revencu, Astrid Eijkelenboom, Claire Bracquemart, Pia Alhopuro, Judith Armstrong, Eulalia Baselga, Claudia Cesario, Maria Lisa Dentici, Melanie Eyries, Sofia Frisk, Helena Gásdal Karstensen, Nagore Gene-Olaciregui, Sirpa Kivirikko, Cinzia Lavarino, Inger-Lise Mero, Rodolphe Michiels, Elisa Pisaneschi, Bitten Schönewolf-Greulich, Ilse Wieland, Martin Zenker, Miikka Vikkula

**Affiliations:** 1 Center for Human Genetics, Cliniques universitaires Saint-Luc, University of Louvain, VASCERN VASCA European Reference Centre, Brussels, Belgium; 2 Department of Pathology, Radboud University Medical Center, VASCERN VASCA European Reference Centre, PO Box 9101, 6500 HB Nijmegen, the Netherlands; 3 Normandie Univ, UNICAEN, Service de Génétique, CHU Caen Normandie, BIOTARGEN EA 7450, VASCERN VASCA European Reference Centre, Caen, 14000 France; 4 HUS Diagnostic Center, Laboratory of Genetics, University of Helsinki and Helsinki University Hospital, VASCERN VASCA European Reference Centre, Helsinki, Finland; 5 Institut de Recerca Sant Joan de Déu, Esplugues de Llobregat, CIBER-ER (Biomedical Network Research Center for Rare Diseases), Instituto de Salud Carlos III (ISCIII), Madrid, and Genomic Unit, Molecular and Genetic Medicine Section, Hospital Sant Joan de Déu, VASCERN VASCA European Reference Centre, Barcelona, Spain; 6 Department of Dermatology, Hospital Sant Joan de Deu, VASCERN VASCA European Reference Centre, Barcelona, Spain; 7 Laboratory of Medical Genetics, Translational Cytogenomics Research Unit, Bambino Gesù Children Hospital and Research Institute, IRCCS, VASCERN VASCA European Reference Centre, Rome, Italy; 8 Medical Genetics Unit, Bambino Gesù Children’s Hospital, IRCCS, VASCERN VASCA European Reference Centre, 00165 Rome, Italy; 9 Sorbonne Université, Département de Génétique, Assistance Publique-Hôpitaux de Paris, Hôpital Pitié-Salpêtrière, VASCERN VASCA European Reference Centre, Paris, France; 10 Department of Molecular Medicine and Surgery, Karolinska Institutet and Department of Clinical Genetics, Karolinska University Hospital, VASCERN VASCA European Reference Centre, Stockholm, Sweden; 11 Department of Genetics, Center of Diagnostics, Copenhagen University Hospital - Rigshospitalet, VASCERN VASCA European Reference Centre, Copenhagen, Denmark; 12 Laboratory of Molecular Oncology, Pediatric Cancer Center Barcelona, Hospital Sant Joan de Déu, VASCERN VASCA European Reference Centre, Barcelona, Spain; 13 Department of Clinical Genetics, HUS Diagnostic Center, University of Helsinki and Helsinki University Hospital, VASCERN VASCA European Reference Centre, Helsinki, Finland; 14 Department of Medical Genetics, Oslo University Hospital, VASCERN VASCA European Reference Centre, Oslo, Norway; 15 Laboratory of Medical Genetics, Translational Cytogenomics Research Unit, Bambino Gesù Children Hospital and Research Institute, IRCCS, VASCERN VASCA European Reference Centre, Rome, Italy; 16 Institute of Human Genetics, University Hospital Otto-Von-Guericke-University, Magdeburg, Germany; 17 Center for Vascular Anomalies, Cliniques Universitaires Saint-Luc, Brussels, Belgium; 18 Human Molecular Genetics , de Duve Institute, University of Louvain, VASCERN VASCA European Reference Centre, Brussels, Belgium; 19 WELBIO Department, WEL Research Institute, Avenue Pasteur, 6, 1300 Wavre, Belgium

**Keywords:** ERN, Gene curation, ISSVA, Mosaic, Precision medicine, Postzygotic, Somatic, VASCERN-VASCA, Vascular malformation

## Abstract

**Background:**

Vascular anomalies caused by somatic (postzygotic) variants are clinically and genetically heterogeneous diseases with overlapping or distinct entities. The genetic knowledge in this field is rapidly growing, and genetic testing is now part of the diagnostic workup alongside the clinical, radiological and histopathological data. Nonetheless, access to genetic testing is still limited, and there is significant heterogeneity across the approaches used by the diagnostic laboratories, with direct consequences on test sensitivity and accuracy. The clinical utility of genetic testing is expected to increase progressively with improved theragnostics, which will be based on information about the efficacy and safety of the emerging drugs and future molecules. The aim of this study was to make recommendations for optimising and guiding the diagnostic genetic testing for somatic variants in patients with vascular malformations.

**Results:**

Physicians and lab specialists from 11 multidisciplinary European centres for vascular anomalies reviewed the genes identified to date as being involved in non-hereditary vascular malformations, evaluated gene–disease associations, and made recommendations about the technical aspects for identification of low-level mosaicism and variant interpretation. A core list of 24 genes were selected based on the current practices in the participating laboratories, the ISSVA classification and the literature. In total 45 gene–phenotype associations were evaluated: 16 were considered definitive, 16 strong, 3 moderate, 7 limited and 3 with no evidence.

**Conclusions:**

This work provides a detailed evidence-based view of the gene–disease associations in the field of vascular malformations caused by somatic variants. Knowing both the gene–phenotype relationships and the strength of the associations greatly help laboratories in data interpretation and eventually in the clinical diagnosis. This study reflects the state of knowledge as of mid-2023 and will be regularly updated on the VASCERN-VASCA website (VASCERN-VASCA, https://vascern.eu/groupe/vascular-anomalies/).

**Supplementary Information:**

The online version contains supplementary material available at 10.1186/s13023-024-03196-9.

## Background

Vascular anomalies comprise a heterogeneous group of disorders, divided into vascular tumours and vascular malformations [1]. The most widely used classification is that of the International Society for the Study of Vascular Anomalies [2], last updated in 2018. In the majority of cases, vascular malformations occur sporadically, but they may also be observed in several individuals in the same family as part of hereditary diseases or syndromes. 

In the last 30 years, significant progress has been made in the elucidation of the genetic causes of vascular malformations. Studies initially focused on the aetiology of the hereditary forms. However, the discovery of somatic *TEK* variants in venous malformation tissues prompted the community to look for such changes in other types of vascular anomalies [3, 4]. The development of deep next-generation sequencing (NGS) techniques, and their increased use due to reduced costs, has facilitated the identification of somatic variants in various types of non-hereditary vascular anomalies. The major pathways involved in hereditary and non-hereditary forms are: 1) the RAS/mitogen-activated protein kinase (MAPK)/extracellular signal-regulated kinase (ERK) pathway, 2) the phosphatidylinositol 3-kinase (PI3K)/protein kinase B (AKT)/mammalian target of rapamycin (mTOR) pathway, and 3) the G-protein coupled receptor signalling pathway [5].

This work focuses on vascular malformations caused by somatic (postzygotic) variants, which represent the majority of patients with vascular malformations. These individuals often manifest unifocal/isolated or segmental vascular malformations. Body asymmetry (overgrowth, less commonly undergrowth) can be associated. The severity varies largely from harmless malformations to complex entities with significant morbidity, such as in CLOVES (congenital lipomatous overgrowth, vascular malformations, epidermal nevi, skeletal/scoliosis and spinal abnormalities), Klippel-Trenaunay syndrome, and Sturge-Weber syndrome. Multiple (multifocal) lesions are sometimes observed, such as in blue rubber bleb nevus syndrome and multifocal sporadic venous malformations, which complicates the diagnosis, as multiple lesions are usually a feature of a hereditary vascular malformation (e.g., capillary malformation–arteriovenous malformation, multiple cutaneous and mucosal venous malformations, cerebral cavernous malformation, etc.).

Pathogenic somatic variants in different genes can be associated with similar phenotypes, such as *TEK* and *PIK3CA* in venous malformations [3, 6, 7]. Likewise, somatic variants in the same gene can be associated with a variety of phenotypes. For instance, somatic variants in *PIK3CA* are associated with isolated lymphatic or venous malformation or with combined slow-flow vascular malformations, collectively known as *PIK3CA*-related overgrowth spectrum (PROS) [8].

Many genetic variants that have been identified as somatic changes in vascular malformations are also known as oncogenic variants in various cancer types. This has led to the idea that – pathway-specific pharmacological inhibitors known as cancer drugs could be repurposed for the treatment of patients with vascular malformations [9, 10] and some of them are already in off-label use or ongoing clinical trials. In addition, Alpelisib was granted accelerated approval from the Food and Drug Administration in 2022 for the treatment of patients aged 2 years or older with severe manifestations of PROS who require systemic therapy. Confirmatory evidence is needed for continued approval [11]. For these novel targeted treatment approaches, a precise molecular diagnosis is mandatory and requires genetic investigations alongside the clinical, radiological and histological assessment. Nonetheless, identifying the causative somatic genetic variants can be challenging, as they are only present in a subset of cells. Indeed, the tissue of a vascular malformation contains a mixture of mutated and non-mutated cells [12]. The percentage of cells carrying the pathogenic variant can be quite low, requiring representative test material and ultra-deep sequencing, which may limit the breadth of the gene panel content. In addition, there is a significant heterogeneity across the approaches currently used by the diagnostic laboratories in terms of technology, gene/exon/variant content, type and depth of sequencing, with direct consequences on the test sensitivity.

The aim of this work is to make recommendations to harmonise and optimise somatic genetic testing of patients with vascular malformations, performed in the participating centres, all members of VASCERN-VASCA or providers of genetic testing for VASCERN-VASCA HCP [13]. The harmonisation mainly focused on a recommended gene content (genes, exons, regions and variants to be covered). Technical aspects for identification of low-level mosaicism were also considered, as well as variant interpretation. Germline genetic testing for hereditary vascular malformations was excluded from this work for two reasons: 1) there is a limited overlap of underlying genetic causes, and 2) the sequencing requirements are different.

## Methods

### Expert panel

The group of experts comprised physicians and lab specialists from 11 multidisciplinary centres, members of the working group on vascular anomalies (VASCA) of the European Reference Network on Multisystemic Vascular Diseases (VASCERN) or providers of genetic testing for VASCERN-VASCA HCP [13]. The expert group included geneticists (clinical and research), dermatologists and lab specialists in molecular genetics, pathology or oncology. Two task forces were formed: clinical and laboratory. The clinical task force was responsible for gene selection and evaluation of the gene–phenotype associations, and the laboratory task force, for selection of the variants and regions of interest, as well as the recommendations concerning the technical aspects (tissue selection, DNA isolation, sequencing techniques and variant interpretation). Virtual meetings of subgroups or the entire group were organised on a regular basis. The work started in 2020 and finished in mid-2023.

### Gene and phenotype selection

Genes of interest in the context of vascular malformations caused by somatic variants were identified through the gene lists of participating expert centres, the ISSVA classification, and a literature review. Only the genes asserted in the literature to be involved in vascular malformations caused by somatic events were considered. Genes involved in hereditary vascular malformations were excluded. If a gene had been reported to be involved both in vascular malformations and vascular tumours (ISSVA classification), the latter association was also assessed (e.g., *GNAQ*). In contrast, genes involved only in vascular tumours were not included (e.g., *GNA14*, *FOS* and *FOSB*). The ISSVA classification was used to define the list of phenotypes. Phenotypes described after the last update of this classification (e.g., *GJA4*-hepatic haemangioma, and *GJA4*-cutaneous venous malformation with cavernous histology) were added.

#### Gene–phenotype association evaluation

The clinical validity of gene-phenotype associations was assessed using a modified framework based on the Gene Clinical Validity Curation Process Standard Operating Procedure version 8 [14], developed by the Clinical Genome Resource Gene Curation Working Group (Table S1). This framework is designed to collect, curate and score the evidence for a gene–disease relationship in two categories of evidence: genetic and experimental. Rules for the assessment and scoring of experimental evidence were adopted from the existing standard operating procedure. In contrast, the assessment of genetic evidence on the basis of case-level data required major adaptations, as the existing framework is not applicable for diseases caused by somatic genetic events. Based on the score, gene-phenotype associations were classified in a five-category system: no evidence (0 points), limited (0,5–5,5 points), moderate (6–11,5 points), strong (12–18 points), and definitive (12–18 points and replication over time).

Data reported in the literature were evaluated. Briefly, a basic score of 0.5 was given for a somatic variant in the respective gene in a case with the respective phenotype, for each curated gene-disease pair. Upgrades of + 0.5 to + 1.5 were given for different levels of evidence for the pathogenicity of the variant: strong prediction of a pathogenic effect by prediction tools: + 0.5; proven functional effect of the variant: + 0.5–1; variant known in ClinVar as likely pathogenic: + 1; and variant known in ClinVar as pathogenic: + 1.5. Additional upgrades of 0.5 to 1 were given if the variant detected in one case affected the neighbouring codons (+ /– 2) or the same codon, respectively. Thereby, a somatic variant of known pathogenic effect, which affects the same codon as another scored variant reaches a maximum total score of 3. At least five independent observations were necessary to reach the maximum score of 12 for genetic evidence. The evaluation matrix is available as supplemental material (Table S1).

For the genetic evidence, the literature was reviewed either comprehensively or until the maximum score was reached (12 points). Total scores for genetic and functional evidence were calculated according to the published standard operating procedures. Based on the total score, a final classification of the clinical validity of a gene–disease association was proposed (no evidence – limited – moderate – strong – definitive). Classification as “definitive” evidence required replication by > 2 publications over time (> 3 yrs.). The evaluation of each gene–disease pair was done by one member of the clinical task force, followed by presentation and discussion in the panel. The final decision on the classification was taken collectively by the clinical task force. The expert panel had the option to maintain or change (upgrade or downgrade) the proposed classification that was based on the scoring system. If the panel decided to change, the reason was documented in the curation summary. This work led to a list of genes for which the assessed evidence for a certain phenotype association supported the consideration as a diagnostic gene.

### Selection of variants, exons and regions of interest

Based on the gene list constituted by the clinical task force, the laboratory task force searched for all reported somatic variants in these genes using the Leiden Open Variation Database (LOVD: [15]) and ClinVar Miner [16] databases, as well as PubMed and Google Scholar with the keywords “*gene name* + vascular malformation”, “*gene name* + vascular anomaly” and “*gene name* + vascular tumour”. Variants were considered somatic if the observed variant allele fraction (VAF) suggested mosaicism or if a variant was present at different allele fractions among different tissue types from the same individual. After the literature and public database search, all members of the laboratory task force were asked to compare their in-house databases with the collected data and complete the list with unreported variants, if any. Variants were then divided into two groups: 1) variants that have been clearly associated with vascular anomalies in the literature and/or databases; and 2) variants that had emerging yet insufficient evidence, as well as variants reported as somatic hotspots in cancer or germline hotspots in RASopathy genes and *TEK*. Cancer hotspot variants were defined through database queries in Catalogue Of Somatic Mutation In Cancer (COSMIC: [17]) and CancerHotspots [18]. Variants identified in the COSMIC database with ≥ 20 documented instances, or in CancerHotspots with ≥ 10 documented instances, were considered potential oncogenic driver variants, in accordance with previous definitions [19–21].

### Technical aspects and variant analysis and interpretation

First, the specific approaches and techniques used by the 11 participating laboratories at the beginning of the work were collected; these included input material, DNA isolation method, sequencing technologies, gene panel content, sequencing platform, bioinformatics pipelines and turn-around time. Based on our experience and a literature review, the laboratory task force drafted analytical workflows that were discussed in the plenary meetings. We provide an expert opinion to improve and standardise laboratory approaches for somatic variant detection and interpretation in the context of vascular malformations caused by postzygotic events.

## Results

### Gene curation

The formal curation process was applied to 24 genes (Table 1 and Supplementary Material 2). In total, 45 gene–phenotype associations were evaluated. Twelve genes were associated with more than one phenotype (vascular malformation or vascular tumour). Of the 45 evaluated gene–phenotype associations, 16 were considered definitive, 16 strong, 3 moderate 7 limited and 3 with no evidence.

Curation matrices for each gene–disease association evaluation are provided as additional file (Supplementary Material 2). There was at least one “definitive” gene–phenotype association for 10 genes (*AKT1*, *GNA11*, *GNAQ*, *IDH1*, *KRAS*, *MAP2K1*, *MAP3K3*, *NRAS*, *PIK3CA* and *TEK*). Out of the 24 genes, 11 were published after the last update of the ISSVA classification (May 2018), of which two have at least one “definitive” association (*KRAS* and *NRAS*), five have at least one “strong” association (*AKT3*, *GJA4*, *HRAS*, *PIK3R1* and *PTPN11*), and four have “limited evidence” (*ARAF*, *CBL*, *GNB2* and *PIK3CD*). When the type of vascular malformation was mixed or not specified in the reports, it was considered under the term of “vascular malformation, various types” (*HRAS*, *NRAS* – not linked to KLA).

**Table 1:**
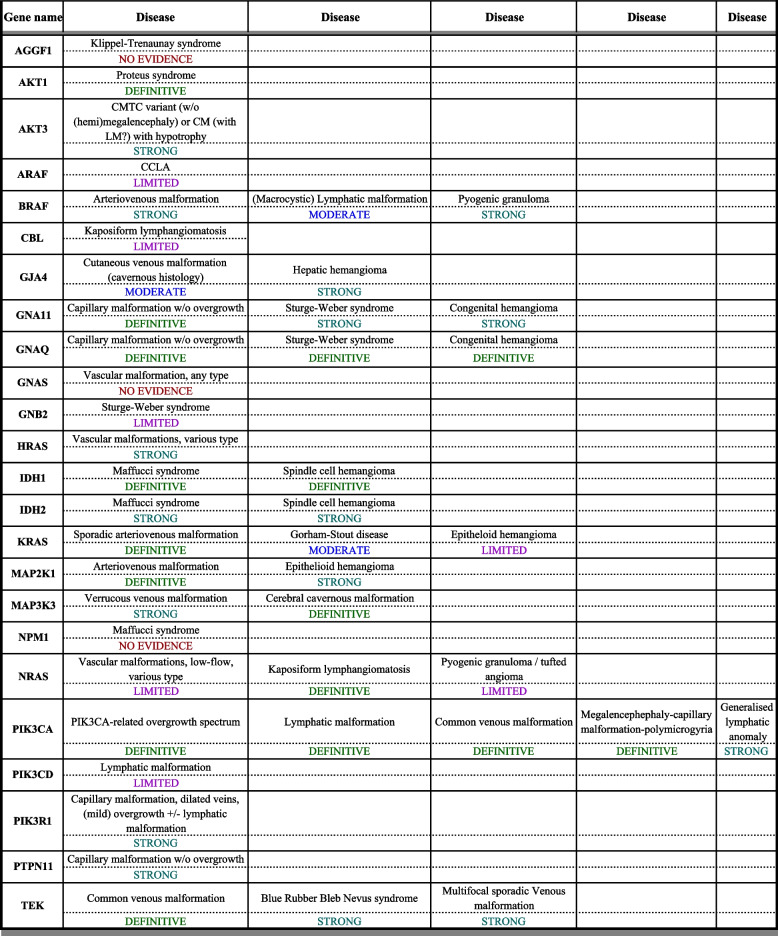
Gene–disease associations and strength of association

### Diagnostic target regions

Based on the review of reported disease-associated variants per curated gene, we defined two levels of target regions per gene to be considered for diagnostic testing of somatic variants in vascular malformations (Table 2 and Table S2). The first level represents the minimum content that should be included in a diagnostic panel (Table 2). This covers pathogenic and likely pathogenic (P/LP) variants reported in the literature and/or databases [15, 16] in patients with non-hereditary vascular anomalies. No additional unreported variants have been identified in the in-house databases from the participating centres. For the delineation of the extended regions of interest, a broader set of variants was accepted, such as variants with insufficient evidence of pathogenicity, and variants in patients with less well-characterised phenotypes (Table S2). This selection also contains: i) areas defined as hotspots in cancer but not yet observed in vascular malformations, and ii) hotspot germline variants in RASopathy genes and *TEK* [22].

**Table 2:** Variants, exons and regions of interest for vascular malformations caused by somatic events

Gene Name	Transcript Reference	Minimal required regions		Optional regions	
		**Exons**	**amino acid residues of interest**	**Exons**	**amino acid residues of interest**
*AKT1*	NM_001382430.1	3	E17	3 4	L52 Q79
*AKT3*	NM_5465.7	3	E17	None	
*ARAF*	NM_001654.5	7	S214	9	R255
*BRAF*	NM_004333.4	15	V600	6 11 14 15 16	T244 to F247, Q257 G466, G469 N581 N581 to K601 Q636
*CBL*	NM_005188.4	15	Y774	2 7 8 9 16	Y102 D359 to K362 Multiple hotspots, splice C404 to R420, splice A877
*GJA4*	NM_002060.3	2	G41	None	
*GNA11*	NM_002067.5	4 5	R183 Q209	None	
*GNA14*	NM_004297.4	4	Q205	None	
*GNAQ*	NM_002072.5	4 5	R183 Q209	2	M59, T96
*GNB2*	NM_005273.4	5	K78	None	
*HRAS*	NM_005343.4	2 3	G12, G13 T58 to M72	3 5	Q61 F156
*IDH1*	NM_005896.4	4	R132	None	
*IDH2*	NM_002168.4	4	R172	4	R140
*KRAS*	NM_004985.5	2 3 4	K5, G12, G13, Q22 Q61, Y64 A146	2 3 4 5	V14, L19 T58 to G60 K117 D153, F156
*MAP2K1*	NM_002755.4	2 3	F53 to E62 P105 to 107, C121 to G128	2 3 5 6 7 8	L42 to D67 E102 to M146 L177 E203 P264, P294 G301, S331
*MAP3K3*	NM_002401.5	13	I441	15	Y544
*NRAS*	NM_002524.5	3 4	Q61 A146	2 3	G12 to P34 T50 to G60
*PIK3CA*	NM_006218.4	Full gene		Full gene	
*PIK3CD*	NM_005026.5	16	L666	13 24	S520 E1021
*PIK3R1*	NM_181523.3	11 13 14	Y452 to Y467 N564 to Y580 W583, splice	9 10 13	R348 G376 N564, K567, splice
*PTPN11*	NM_002834.5	3 12 13	E76 V428, A461 T507	1_13	Multiple hotspots
*TEK*	NM_00459.5	17 22 23	Y897 to A925 R1099X T1105 to G1115	15 17 18 23	R849 L920 F960 K1100 to Y1113

This table describes regions of interest (genes, exons, amino acids) that should be studied for somatic exploration of vascular anomalies. The regions of interest are divided into two groups: “Minimal required regions” and “Optional regions”. The first one represents what is well described and implicated in the pathogenicity of somatic vascular anomalies, and the second one represents hotspot variants reported in cancer, or germline hotspots in *RASopathy genes* and *TEK*, with insufficient evidence of pathogenicity in non-hereditary vascular anomalies. Reference transcripts are from ensembl.org. Data are from literature and databases (LOVD, Clinvar Miner, Cosmic (v96) and CancerHotspots (v2))

### Technical aspects

The detection of somatic variants has special technical requirements. The approaches and techniques, from sample collection to DNA extraction and sequencing technologies, used by the 11 laboratories of the participating centres at the beginning of this study are listed anonymously in Table S3.

### Tissue selection and DNA requirements

In participating centres, sequencing analysis for somatic variants was preferably performed on DNA from affected tissue, as the variants are usually absent or present at only very low allele fraction in other sources, such as blood or saliva. Affected tissue samples were obtained by lesional biopsies or from surgical resections. They were further processed in various ways until DNA extraction, mainly as native tissue samples in saline solution, frozen or in RNAlater solution, but some laboratories also used formalin-fixed paraffin embedded (FFPE) tissue (Table S3). The specifics of tissue processing were matched to the laboratory protocols for subsequent analysis (for example, not all the library preparation methods are compatible with genomic DNA (gDNA) from FFPE tissue). For most centres, histological examination was considered in the diagnostic work-up in a pathology lab, especially if the diagnosis was unclear or to ensure that the biopsy is representative (deep malformation). Some laboratories performed tissue dissection (removing unaffected tissue, e. g. epidermidis) to increase the sensitivity of somatic testing (Table S3).

#### Minimum tissue requirements

The minimum amount of tissue was dependent on the input criteria (quantity and quality) of the laboratory protocols for subsequent analysis. A punch or excisional biopsy size of 4 mm was usually sufficient for subsequent analysis in the laboratory’s protocols.

#### DNA isolation methods

A variety of commercial tissue DNA extraction kits and protocols were used by the participating laboratories to isolate gDNA from the affected tissue. The specifics of the DNA isolation protocols were adapted according to both the tissue preparation and the laboratory protocols for subsequent analysis. The minimum DNA input for analysis ranged from 5 to 200 ng (Table S3). Quality control of gDNA were included in each laboratory protocol depending on the methods used for sequencing part.

### Sequencing analysis by NGS

Most centres were using custom targeted panels, while three centres (9, 10 and 11) were using whole-exome sequencing (WES) and applied a virtual in silico panel of genes of interest for the analysis of WES data (Table S3). Two laboratories were using unique molecular identifiers (UMIs) to increase sequencing accuracy. For those centres using WES, the analysed gene panels also included genes for hereditary vascular malformations, to enable germline and somatic testing in the same analysis flow. Higher read depth was used (120X-400X minimal read depth) compared to standard germline WES testing. However, the target regions may have suboptimal coverage compared to custom panels and low-frequency variants can thereby go undetected. Minimal sequencing depth for custom panels ranged from 100 to 2000X (Table S3).

#### Read alignment and variant detection

In house pipelines as well as commercial pipelines were used by the participating centres to align and call the variants (Table S3). Bioinformatics pipelines were adapted to the special requirements of somatic variant calling (with a low VAF).

#### Technical sensitivity and specificity

Pathogenic variants that are detected in vascular malformations caused by somatic events are generally present only in a subset of cells and the mutant allele frequency in DNA from mixed lesional tissue samples may be quite low. In the participating diagnostic routine laboratories, the indicated cut off for the minimum VAF varied from 1 to 3% (Table S3). If depth for detecting low VAF is not reached due to technical limitations, it will reduce the analytical sensitivity of the assay. Notably, approximately 15% (*n* = 41/262) of cases with a pathogenic variant in *PIK3CA* detected by the members of this working group had a VAF of < 3% (internal data from the participating centres).

Each laboratory established empirically its sequencing requirements, such as the minimum sequencing depth, a minimum number of variant reads and the minimum VAF to be reported (Table S3). Binomial distribution-based coverage calculation can aid in determining the minimum sequencing depth [23]. UMI-based sequence analysis was successfully used by two of the participating laboratories to increase test accuracy (Table S3). One laboratory used strand-specific amplification, which may help to discriminate genuine C:G > T > A variants from deamination artefacts in FFPE samples. If tissue handling and NGS library flows are combined with the processing of solid tumour specimens, there is a possibility of cross-contamination from solid tumour specimens as the origin of pathogenic variants with a low VAF. One laboratory added common single nucleotide polymorphisms (SNPs) in the library preparation to trace this cross-contamination during analysis of sequences.

### Alternative methods of somatic variant detection/validation

Some laboratories used digital PCR (dPCR)/droplet digital PCR (ddPCR) to confirm low frequency variants or detect recurrent hotspots variants in genes such as *GNAQ*, *GNA11*, *PIK3CA*, and *TEK*.

### Variant interpretation

#### Variant filtering

As usually performed in NGS-based analysis, common benign variants and artefacts were removed from the detected list of variants prior to interpretation.

#### Variant classification and reporting

All centres indicated that their classification of variant pathogenicity was essentially based on ACMG recommendations [24]. The AMP/ACMG/ASCO recommendations were used by the centres to report somatic test results for vascular malformations [25]. All the information required for unambiguous clinical interpretation of the test result was reported according to the guidelines. The Human Genome Variation Society (HGVS) standard nomenclature [26] providing the transcript reference sequence was used to annotate all detected variants. Coverage of the NGS assay, VAF, exons/hotspots that do not meet the quality standards, description of methods, assay performance characteristics, limitations of the assays used and a list of genes covered are the minimal data content indicated in reports [25]. All reported variants were classified as described above, and pathogenic and likely pathogenic alterations were generally disclosed. Unexpected findings or variants of unknown significance were discussed with clinicians prior to reporting. Recommendations for any supplemental testing were made when appropriate.

## Discussion

The aim of this work was to propose a performant and accurate genetic testing approach for patients with vascular malformations caused by somatic genetic events. At the beginning of this work, there was a large heterogeneity between the centres in the VASCERN-VASCA regarding the genes and the exonic regions that were screened, as well as in the methodological approaches. Therefore, our first aim was to harmonise the content of the diagnostic gene panel, by proposing a core gene list based on evidence of associations with vascular malformations. Our second aim was to make recommendations for the technical approaches.

### Scope of diagnostic somatic testing in vascular malformations

By systematic evaluation of gene-disease associations, we came up with a list of 24 genes, for which there was usable published evidence for their role in vascular malformations driven by somatic variants. For a subset of 17 genes the evidence was rated “strong” or “definitive” for at least one gene-phenotype association. We consider these core genes to be a mandatory content of any diagnostic panel used for a broad spectrum of vascular malformations. We also recommend the inclusion of a further 4 genes with “limited evidence”, as it can be expected that these genes may be upgraded as new published observations emerge. In contrast, we do not recommend the inclusion of the remaining 3 genes, for which “no evidence” of an association with vascular malformations was found (Table 1).

Since somatic (gain-of-function) variants that are associated with vascular malformations are typically restricted to specific hotspot regions within the respective genes, we also aimed to provide recommendations on variants and regions of interest for each gene. The sites of variants reported in vascular malformations should be covered by a diagnostic test as a minimum requirement (Table 2). As the variants associated with vascular malformations overlap with recurrent oncogenic variants found in cancer in the respective genes, a broader definition of the regions of interest also includes these (Table S2). In general, laboratories using targeted gene panels that only partially cover genes according to their defined hotspots should be aware that novel regions of interest may rapidly emerge.

Notably, the list of genes evaluated in this work is significantly larger than the number of genes mentioned in the last ISSVA classification, illustrating the major progress that has been made since the last update (May 2018). There are 11 new genes (*AKT3*, *ARAF*, *CBL*, *GJA4*, *GNB2*, *HRAS*, *KRAS*, *NRAS*, *PIK3CD*, *PIK3R1* and *PTPN11*) that are not in the ISSVA classification (2018). Of these, seven have at least one “definitive” (*KRAS* and *NRAS*) or “strong” (*AKT3*, *GJA4*, *HRAS*, *PIK3R1* and *PTPN11*) gene–phenotype association. The remaining four genes (*ARAF*, *CBL, GNB2* and *PIK3CD*) have been associated with vascular malformations with “limited evidence”. For *ARAF*, two patients have been reported by the same group in 2019 [27] with lymphangiomatosis or central conducting lymphatic anomaly, respectively. Based on the available data, it is not clear whether both variants were somatic or not. The observation of *ARAF* variants in lymphatic malformations has not been replicated since. Case reports have been published for *GNB2, CBL* and *PIK3CD*, [28–30] describing patients with Sturge-Weber syndrome (*GNB2*) or lymphatic anomalies (*CBL* and *PIK3CD*). Further evidence is needed to confirm the involvement of these genes in vascular malformations. In the most recent version of the ISSVA classification, the *NPM1* gene was indicated as being associated with Maffucci syndrome. However, a careful review of the literature does not support this association, as the only patient with a variant in this gene reported to date was a 7-year-old girl with Maffucci syndrome and acute myeloid leukaemia [31]. This patient had two somatic variants, both in the leukemic cells and in the vascular lesion: a frameshift variant in the *NPM1* gene and a missense variant in the *IDH1* gene. As *IDH1* is strongly associated with Maffucci syndrome, the variant identified in this gene can explain this part of the phenotype. Somatic *NPM1* variants are instead associated with acute myeloid leukaemia (OMIM **#**601,626). Thus, we conclude that the *NPM1* variant is not involved in the pathogenesis of Maffucci syndrome.

There is also increasing evidence that several genes (particularly the core genes of the RAS-MAPK and PI3K-AKT cascades) are involved in a wider range of vascular anomalies than previously recognised, probably reflecting variability in the timing of the mutation, the cell type affected and other factors. For example, the E542K variant in *PIK3CA* has been identified in isolated LM, isolated VM and PROS. New phenotypes were added for a total of five genes: *BRAF* with sporadic AVM (strong evidence) and LM (moderate evidence), *GNA11* with Sturge-Weber syndrome (strong evidence), *MAP2K1* with epithelioid haemangioma (strong evidence), *MAP3K3* with cerebral cavernous malformation (definitive evidence) and *PIK3CA* with generalised lymphatic anomaly (strong evidence).

We evaluated gene–disease associations until variant level, when relevant. As mentioned above, the same pathogenic variants can be associated with different or overlapping phenotypes and with different extension and severity of the disease. However, different variants in the same gene can also be associated with specific phenotypes; for example, the R183Q variant in *GNAQ* has been associated with CM and Sturge-Weber syndrome, but not with non-involuting congenital haemangiomas (NICH) or rapid-involuting congenital haemangiomas (RICH), which are instead associated with the Q209H/L/P variants.

The expanding genetic heterogeneity and phenotypic diversity associated with individual genes challenges the use of narrowly focused diagnostic assays that include only one or a small panel of genes targeted to a defined clinical phenotype. However, the predominance of recurrent variants in some distinctive phenotypes still leaves a possible rationale for the use of targeted methods such as digital PCR, which can be an efficient and cost-effective first-tier approach to search for highly recurrent hotspot variants or to analyse genes with a narrow mutational spectrum, such as *GNAQ, GNA11*, *PIK3CA* and *TEK* hotspot variants. However, negative results from such an approach, should be followed by testing of a more comprehensive gene panel.

Genes involved in vascular malformations caused by somatic events may also be analysed in combination with genes involved in hereditary vascular malformations (germline events), although this is not specifically recommended. Instead, the expert panel agreed that the analysis of genes involved in hereditary vascular malformations is generally not essential in sporadic patients with a type of vascular malformation that is usually caused by somatic events, as the underlying genes are mostly different, and the clinical presentation can usually guide towards somatic or germline testing, respectively. Moreover, for laboratories using custom panels, the inclusion of the genes for hereditary forms would greatly affect the size of the panel, which may conflict with the need for very deep sequencing to detect somatic variants with very low VAFs. The combination of germline and somatic testing in one assay should take into account cost-effectiveness, which also depends on the local laboratory equipment and organisation. Nevertheless, the limitation to a very strictly-defined gene content will become obsolete with the advent (and cost efficiency) of new, very large-scale sequencing technologies. For laboratories using a WES-based approach it is easy to open their analysis to the genes involved in hereditary vascular malformations based on the patient’s phenotype (e.g. *KRIT1* for patients with hyperkeratotic cutaneous capillary‐venous malformations, and *RASA1, EPHB4, ENG1, ACVRL1, SMAD4, PTEN* for AVM). However, it has to be noted that WES is usually not performed at a sequencing depth that is sufficient to reach a reliable mosaic detection threshold of 1% VAF. For laboratories using a custom panel, on the other hand, a low threshold for extending the analysis toward the genes involved in hereditary forms can be suggested, if the phenotype is compatible with such a disease. It has to be noted, that multiplicity of vascular lesions or a positive family history are not always present in hereditary vascular malformations. If somatic testing is performed exclusively, potentially pathogenic germline variants should be reported and the patient referred for genetic counselling and germline testing.

Somatic variants in overlapping groups of genes may also be involved in other disorders, for example genes of the PI3K-AKT-mTOR pathway in segmental overgrowth or brain malformations or genes of the RAS-MAPK pathway in neurocutaneous mosaic disorders. Thus, laboratories may find it more useful and efficient to use a combined panel rather than separate panels to test for these groups of conditions.

### Technical recommendations for genetic testing of somatic variants

Recommendations for technical aspects of somatic variant testing are summarised in Table 3. The recommendations represent a consensus of expert opinion based on the practice and experience of the reference centres involved in this study, and are intended to assist clinical laboratories in the detection of somatic variants and to ensure a high quality of sequencing results.

**Table 3:** Technical recommendations for genetic testing of somatic variants

Technical aspect	Recommendation of the VASCERN-VASCA expert group
Tissue sampling	4 mm of affected tissue specimen from debulking/reduction surgery, or skin biopsy of affected tissue containing abnormal vasculature is the minimum tissue size recommended for adequate DNA yield Fresh/frozen and FFPE samples can be considered Blood samples are generally not recommended, as the diagnostic yield is low. Cell-free DNA from blood plasma or lymphatic fluid are a promising alternative source, but need further evaluation
Histologic examination	Recommended for differential diagnostics and/or estimation of the representativeness of the sample. Dissection (removal of epidermis) can be performed to increase the percentage of affected tissue
Tissue culturing	Tissue culturing is not necessary, but culturing endothelial cells from affected tissue may aid subsequent variant detection
DNA extraction	Standard DNA extraction protocols
DNA quality	Low-quality samples can be analysed; however, it reduces sensitivity and may introduce sequencing artefacts. Cost-effectiveness of prior DNA quality control must be determined in each laboratory
Gene-disease validity	Core gene list (Table 1) and updates thereof https://vascern.eu/
Expected VAFs	Mosaic variants can be present at multiple frequencies, expected VAFs are mostly lower than 10%
Target region	Minimum required regions that must be covered for the detection of somatic variants associated with vascular malformation are reported in Table 2. Recommended optional regions are also listed in Table S2
Sequencing analysis by NGS	Various platforms can be used UMI-based sequencing is recommended especially for amplicon-based, but also for hybridisation-based capture technologies. For the latter, if UMIs are not used, read deduplication is recommended, which can be based on read start and end coordinates; Strand-specific amplification or capture can help to discriminate genuine C:G > T > A variants from deamination artefacts in FFPE samples
Technical sensitivity and specificity	Sequencing depth and minimum number of variant reads should be empirically defined. Detection threshold of 1% is recommended. Including common SNPs in the library preparation is advised to detect cross-contamination from tumour samples when handled in parallel
Read alignment, variant detection, and variant filtering	Diagnostically validated pipelines for somatic calling are recommended. Prefiltering is advised to remove benign variants and artefacts
Validation of NGS findings	Recommended for all variants with VAF less than 5% using dPCR or a second NGS analysis
Alternative methods of somatic variant detection	dPCR can be used for the detection of recurrent variants associated with specific phenotypes
Variant classification	ACMG classification with modifications suggested in PMID: 35,997,716 and PMID: 34,040,190 are recommended. Literature/database searches should extend beyond cancer variant data annotation and interpretation
Reporting	AMP/ACMG/ASCO recommendations are largely applicable (PMID: 27,993,330). Variants should be annotated using the HGVS standard nomenclature. Reports should provide VAF, coverage, regions that do not meet quality standards, description of methods, limitations and list of genes analysed. Pathogenic/likely pathogenic variants should be disclosed. Recommendations for supplemental testing should be made, such as germline panel testing, based on the indication and clinical context
Identification of potential germline variants	Potential pathogenic/likely pathogenic germline variants should be included in the report and referral for germline testing and genetic counselling should be advised
Data sharing	Submission of variants with phenotypic, clinical significance, and classification criteria to DNA variant databases, such as ClinVar and LOVD, is recommended to increase knowledge of the genetics of vascular anomalies

Regarding the source of DNA for diagnostic testing, a tissue sample containing abnormal vasculature is the material of choice and gold standard. Since surgical resection tissue is not always available, a skin biopsy with a punch from the site of the lesion can be obtained. Cell culturing from tissue samples is not routinely necessary, but can help increase the VAF if endothelial cells from the affected tissue are isolated and used for culture [32, 33]. Blood leucocyte DNA is not recommended as the diagnostic yield is relatively low [34], with a few exceptions, such as for some patients with MCAP [35] or MSVM (multiple sporadic venous malformations) [36]. Depending on the phenotype, other non-invasive specimens, such as urine (urinary epithelial cells) [37] or saliva (buccal cells) [38] may also contain the causative variant, but their use cannot be recommended for the general routine. Cell-free DNA (cfDNA) analysis is emerging as an attractive alternative to tissue-based testing. This would be particularly beneficial when an invasive procedure is not possible and for young patients. Nevertheless, the VAF of disease-causing variants in cfDNA is usually much lower than in lesional tissue DNA (often less than 1%) and requires highly sensitive techniques, such as dPCR [39, 40]. The diagnostic yield of cfDNA in vascular malformations is currently rather low. It may be increased by using cfDNA from an efferent vein [41] or lymphatic fluid [40]. However, the use of cfDNA for diagnostic purposes needs to be further developed and validated.

Low VAFs of the disease-causing somatic variants in mixed tissue specimens or other diagnostic samples from patients with vascular malformations present a major diagnostic challenge. The VAFs of somatic driver variants are generally lower than those typically found in solid tumours. Therefore, routine settings adopted from molecular oncology may be insufficient for vascular malformations. In large patient cohorts, most pathogenic variants are present in lesional tissue samples with a VAF less than 10%, even in patients with severe phenotypes [42, 43]. The expected VAF range depends on the sample type (e.g. [34] and clinical context (e.g. [44] and may even fall below 1%. Enrichment of mutant cells in a given sample might be achieved by tissue dissection, microdissection, endothelial cell culturing or cell separation [45, 46]. These approaches may be considered when faced with a negative result. However, the current experience is insufficient to recommend their routine use. In samples of poor DNA quality or quantity, the sensitivity for low-level mosaicism is reduced and the probability of ambiguous results is increased. The expert group recommends to include a quality control of the gDNA starting material to relate the DNA quality assessment with the sequencing results. If tissue handling and NGS library flows are combined with the processing of solid tumour specimens, there is possibility of cross-contamination from solid tumour specimens as the origin of pathogenic variants with a low VAF. Different methods using polymorphic tags in the library preparation and/or post-sequencing bio-informatic analysis can be applied to detect such cross-contamination [47, 48].

Concerning the sensitivity threshold for somatic variants in lesional tissue samples, the expert group recommendation for the minimum VAF is set at 1%. Sequencing depth and minimum number of variant reads needed to reach this threshold should be empirically defined for each work-flow. Standardized set of DNA samples carrying mosaic variants with different allele fractions are made available by companies helping in panel development. It is also possible to exchange DNA samples between laboratories for genetic tests that are not yet covered by an external quality assessment scheme. Binomial distribution-based coverage calculations can be used to determine the probability to detect a variant at a certain allele ratio [23]. Notably, increasing sequencing depth of libraries, especially those derived from poor quality material, per se has limited effect on sensitivity because of the background level of technical artefacts that are inherent to the technology. Artefacts can arise from different sources, including tissue fixation with formalin, PCR and sequencing; they are therefore dependent on sample type, library preparation and sequencing technique [49]. When FFPE tissue samples are used, common base substitution artefacts, particularly C > T/G > A caused by cytosine deamination, significantly limit the detection threshold [50]. An elegant option to overcome some of these limitations of current NGS technologies and thereby lower the detection threshold is the use of UMI-based sequencing. These are complex molecular barcodes that are added to DNA prior to any PCR amplification steps. This enables the definition of the consensus sequence of PCR multiplicates derived from a single template DNA molecule, thereby eliminating amplification and sequencing artefacts [51]. The use of UMIs is recommended especially for amplicon-based, but also for hybridisation-based capture technologies. For the latter, if UMIs are not used, read deduplication is recommended, which can be based on read start and end coordinates.

Generally, ambiguous findings and results close to the detection threshold of the given test should be interpreted with caution in the context of the clinical indication, and independent confirmation should be sought. This can preferably be achieved by an alternative technique, such as dPCR, or if not available, by repeating the NGS analysis [52]. Digital PCR provides a high depth of genotyping allowing the detection of specific variants at VAFs as low as 0.1%, with a minimal gDNA input requirements (5 ng of gDNA per test) and can also be used if the gDNA quality or quantity is suboptimal (e.g. DNA from FFPE tissue). The use of dPCR as a first-tier test for specific indications is discussed above. The need for specific assays for each change at the nucleotide level and limited multiplexing capabilities constrain the use of dPCR in vascular malformation diagnostics. The use of blocker displacement amplification as an alternative method to confirm variants with very low VAF in vascular malformations has recently been reported [46].

Bioinformatic pipelines for NGS data analysis need to be adapted for the specific needs of low-level mosaic detection. For variant callers the threshold may need to be adjusted for the desired minimum calling VAF. In addition, for some recurrent hotspot positions, it may be considered to program a systematic variant calling and/or to manually inspect the aligned sequences in a genome viewer such as integrative genomics viewer (IGV: [53]). On the other hand, a more agnostic approach with an exploratory setting based on deep WES may be useful for detecting disease causing variants in novel genes [54].

The interpretation and classification of causal somatic variants in vascular malformations can sometimes be challenging. Although disease-causing variants in several genes largely overlap with oncogenic drivers detected in solid tumours, other variants in specific genes can be found (almost) exclusively in vascular malformations (e.g., variants in the receptor tyrosine kinase *TEK* gene have not been reported in cancer). Furthermore, atypical variants in some oncogenes are (almost) exclusively found in vascular malformations (e.g. atypical *HRAS* and *KRAS* indel variants [55]).

In our experience, the American College of Medical Genetics/Association for Molecular Pathology (ACMG/AMP) general criteria of classification of germline variants [24] were not completely applicable for somatic variant classification. Other classification guidance schemes from oncology, such as the joint consensus recommendations of AMP, ACMG, American Society of Clinical Oncology (ASCO) [25, 56] and European Society for Medical Oncology (ESMO) [57] can be used, but were found to focus on clinical relevance and to lack guidance on variant classification. An exception was the Belgian ComPerMed, which proposed guidelines for biological classification and clinical interpretation of somatic variants [58]. Gene-specific recommendations were recently reported for *PIK3R1* [21], and for *AKT3*, *MTOR*, *PIK3CA* and *PIK3R2* by the ClinGen Brain Malformation Variant Curation Expert Panel [59]. Such gene specific recommendations should be tested and validated for vascular malformations.

## Conclusion

Based on the VASCERN-VASCA expertise, our aim was to optimise and guide the diagnostic genetic testing performed for non-hereditary vascular malformations caused by somatic variants. We defined the core gene list that should be tested and evaluated, the level of evidence of the gene–phenotype associations and the variants/regions of interest that should be included in a (virtual) panel. Knowing the gene-phenotype relationship and the strength of this association can greatly help laboratories to interpret the data and contribute to clinical diagnosis. This study reflects the state of knowledge as of mid-2023 and will be regularly updated on the VASCERN-VASCA website [13]. Laboratories around the world should be able to use these data.

## Supplementary Information

**Supplementary Material 1.**

**Supplementary Material 2.**

**Supplementary Material 3.**

**Supplementary Material 4.**

## Data Availability

All data analysed during this study are included in the article and its supplementary information files.

## Abbreviations

ACMG: American College of Medical Genetics
AMP: Association for Molecular Pathology
ASCO: American Society of Clinical Oncology
cfDNA: Cell-free DNA
ESMO: European Society for Medical Oncology
gDNA: Genomic DNA
COSMIC: Catalogue Of Somatic Mutation In Cancer
CLOVES: Congenital lipomatous overgrowth, vascular malformations, epidermal nevi, skeletal/scoliosis and spinal abnormalities
dPCR: Digital PCR
ddPCR: Droplet digital PCR
HGVS: Human Genome Variation Society
ISSVA: International Society for the Study of Vascular Anomalies
IGV: Integrative genomics viewer
LOVD: Leiden Open Variation Database
MCAP: Megalencephaly-capillary malformation
MSVM: Multiple sporadic venous malformations
NGS: Next-generation sequencing
NICH: Non-involuting congenital haemangiomas
PHTS: *PTEN* Hamartoma tumour syndrome
PROS: PIK3CA-related overgrowth spectrum
SNP: Single-nucleotide polymorphism
RICH: Rapidly involuting congenital haemangiomas
VAF: Variant allele fraction
UMI: Unique molecular identifier
WES: Whole-exome sequencing

## References

[1] Mulliken JB, Glowacki J. Hemangiomas and vascular malformations in infants and children: a classification based on endothelial characteristics. Plast Reconstr Surg. 1982;69(3):412–22. 7063565 10.1097/00006534-198203000-00002

[2] International Society for the Study of Vascular Anomalies. ISSVA classification for vascular anomalies. Revised 2018. Available from: https://www.issva.org/UserFiles/file/ISSVA-Classification-2018.pdf.

[3] Limaye N, Wouters V, Uebelhoer M, Tuominen M, Wirkkala R, Mulliken JB, et al. Somatic mutations in angiopoietin receptor gene TEK cause solitary and multiple sporadic venous malformations. Nat Genet. 2009;41(1):118–24. 19079259 10.1038/ng.272 PMC2670982

[4] Limaye N, Boon LM, Vikkula M. From germline towards somatic mutations in the pathophysiology of vascular anomalies. Hum Mol Genet. 2009;18(R1):R65–74. 19297403 10.1093/hmg/ddp002 PMC2657941

[5] Queisser A, Seront E, Boon LM, Vikkula M. Genetic Basis and Therapies for Vascular Anomalies. Circ Res. 2021;129(1):155–73. 34166070 10.1161/CIRCRESAHA.121.318145

[6] Limaye N, Kangas J, Mendola A, Godfraind C, Schlogel MJ, Helaers R, et al. Somatic Activating PIK3CA Mutations Cause Venous Malformation. Am J Hum Genet. 2015;97(6):914–21. 26637981 10.1016/j.ajhg.2015.11.011 PMC4678782

[7] Castel P, Carmona FJ, Grego-Bessa J, Berger MF, Viale A, Anderson KV, et al. Somatic PIK3CA mutations as a driver of sporadic venous malformations. Sci Transl Med. 2016;8(332):332ra42. 27030594 10.1126/scitranslmed.aaf1164 PMC4962922

[8] Canaud G, Hammill AM, Adams D, Vikkula M, Keppler-Noreuil KM. A review of mechanisms of disease across PIK3CA-related disorders with vascular manifestations. Orphanet J Rare Dis. 2021;16(1):306. 34238334 10.1186/s13023-021-01929-8 PMC8268514

[9] Nguyen HL, Boon LM, Vikkula M. Vascular Anomalies Caused by Abnormal Signaling within Endothelial Cells: Targets for Novel Therapies. Semin Interv Radiol. 2017;34(3):233–8. 10.1055/s-0037-1604296 PMC5615384 28955112

[10] Van Damme A, Seront E, Dekeuleneer V, Boon LM, Vikkula M. New and Emerging Targeted Therapies for Vascular Malformations. Am J Clin Dermatol. 2020. 10.1007/s40257-020-00528-w 32557381

[11] FDA accelerated approval for Alpelisib. Available from: https://www.fda.gov/drugs/resources-information-approved-drugs/fda-approves-alpelisib-pik3ca-related-overgrowth-spectrum.

[12] Nguyen HL, Boon LM, Vikkula M. Genetics of vascular malformations. Semin Pediatr Surg. 2014;23(4):221–6. 25241102 10.1053/j.sempedsurg.2014.06.014

[13] VASCERN-VASCA. Available from: https://vascern.eu/groupe/vascular-anomalies/.

[14] Gene Clinical Validity Curation Process Standard Operating Procedure version 8. Available from: https://clinicalgenome.org/docs/summary-of-updates-to-the-clingen-gene-clinical-validity-curation-sop-version-8/.

[15] LOVD: Leiden Open Variation Database. Available from: https://www.lovd.nl/.

[16] ClinVar Miner database. Available from: https://clinvarminer.genetics.utah.edu/.

[17] COSMIC: Catalogue Of Somatic Mutation In Cancer. Available from: https://cancer.sanger.ac.uk/cosmic.

[18] CancerHotspots database. Available from: https://www.cancerhotspots.org/#/home.

[19] Chang MT, Bhattarai TS, Schram AM, Bielski CM, Donoghue MTA, Jonsson P, et al. Accelerating Discovery of Functional Mutant Alleles in Cancer. Cancer Discov. 2018;8(2):174–83. 29247016 10.1158/2159-8290.CD-17-0321 PMC5809279

[20] Walsh MF, Ritter DI, Kesserwan C, Sonkin D, Chakravarty D, Chao E, et al. Integrating somatic variant data and biomarkers for germline variant classification in cancer predisposition genes. Hum Mutat. 2018;39(11):1542–52. 30311369 10.1002/humu.23640 PMC6310222

[21] Cottrell CE, Bender NR, Zimmermann MT, Heusel JW, Corliss M, Evenson MJ, et al. Somatic PIK3R1 variation as a cause of vascular malformations and overgrowth. Genet Med. 2021;23(10):1882–8. 34040190 10.1038/s41436-021-01211-z PMC8486672

[22] Gelb BD, Cave H, Dillon MW, Gripp KW, Lee JA, Mason-Suares H, et al. ClinGen’s RASopathy Expert Panel consensus methods for variant interpretation. Genet Med. 2018;20(11):1334–45. 29493581 10.1038/gim.2018.3 PMC6119537

[23] Eijkelenboom A, Kamping EJ, Kastner-van Raaij AW, Hendriks-Cornelissen SJ, Neveling K, Kuiper RP, et al. Reliable Next-Generation Sequencing of Formalin-Fixed, Paraffin-Embedded Tissue Using Single Molecule Tags. J Mol Diagn. 2016;18(6):851–63. 27637301 10.1016/j.jmoldx.2016.06.010

[24] Richards S, Aziz N, Bale S, Bick D, Das S, Gastier-Foster J, et al. Standards and guidelines for the interpretation of sequence variants: a joint consensus recommendation of the American College of Medical Genetics and Genomics and the Association for Molecular Pathology. Genet Med. 2015;17(5):405–24. 25741868 10.1038/gim.2015.30 PMC4544753

[25] Li MM, Datto M, Duncavage EJ, Kulkarni S, Lindeman NI, Roy S, et al. Standards and Guidelines for the Interpretation and Reporting of Sequence Variants in Cancer: A Joint Consensus Recommendation of the Association for Molecular Pathology, American Society of Clinical Oncology, and College of American Pathologists. J Mol Diagn. 2017;19(1):4–23. 27993330 10.1016/j.jmoldx.2016.10.002 PMC5707196

[26] HGVS standard nomenclature. Available from: http://www.hgvs.org.

[27] Li D, March ME, Gutierrez-Uzquiza A, Kao C, Seiler C, Pinto E, et al. ARAF recurrent mutation causes central conducting lymphatic anomaly treatable with a MEK inhibitor. Nat Med. 2019;25(7):1116–22. 31263281 10.1038/s41591-019-0479-2

[28] Fjaer R, Marciniak K, Sundnes O, Hjorthaug H, Sheng Y, Hammarstrom C, et al. A novel somatic mutation in GNB2 provides new insights to the pathogenesis of Sturge-Weber syndrome. Hum Mol Genet. 2021;30(21):1919–31. 34124757 10.1093/hmg/ddab144 PMC8522634

[29] Foster JB, Li D, March ME, Sheppard SE, Adams DM, Hakonarson H, et al. Kaposiform lymphangiomatosis effectively treated with MEK inhibition. EMBO Mol Med. 2020;12(10): e12324. 32894644 10.15252/emmm.202012324 PMC7539180

[30] Wang S, Wang W, Zhang X, Gui J, Zhang J, Guo Y, et al. A somatic mutation in PIK3CD unravels a novel candidate gene for lymphatic malformation. Orphanet J Rare Dis. 2021;16(1):208. 33964933 10.1186/s13023-021-01782-9 PMC8106842

[31] Akiyama M, Yamaoka M, Mikami-Terao Y, Ohyama W, Yokoi K, Arakawa Y, et al. Somatic mosaic mutations of IDH1 and NPM1 associated with cup-like acute myeloid leukemia in a patient with Maffucci syndrome. Int J Hematol. 2015;102(6):723–8. 26508204 10.1007/s12185-015-1892-z

[32] Huang L, Couto JA, Pinto A, Alexandrescu S, Madsen JR, Greene AK, et al. Somatic GNAQ Mutation is Enriched in Brain Endothelial Cells in Sturge-Weber Syndrome. Pediatr Neurol. 2017;67:59–63. 27919468 10.1016/j.pediatrneurol.2016.10.010 PMC5303551

[33] Nikolaev SI, Vetiska S, Bonilla X, Boudreau E, Jauhiainen S, Rezai Jahromi B, et al. Somatic Activating KRAS Mutations in Arteriovenous Malformations of the Brain. N Engl J Med. 2018;378(3):250–61. 29298116 10.1056/NEJMoa1709449 PMC8161530

[34] Lalonde E, Ebrahimzadeh J, Rafferty K, Richards-Yutz J, Grant R, Toorens E, et al. Molecular diagnosis of somatic overgrowth conditions: A single-center experience. Mol Genet Genomic Med. 2019;7(3): e536. 30761771 10.1002/mgg3.536 PMC6418364

[35] Mirzaa G, Timms AE, Conti V, Boyle EA, Girisha KM, Martin B, et al. PIK3CA-associated developmental disorders exhibit distinct classes of mutations with variable expression and tissue distribution. JCI Insight. 2016;1(9). 10.1172/jci.insight.87623 PMC5019182 27631024

[36] Soblet J, Kangas J, Natynki M, Mendola A, Helaers R, Uebelhoer M, et al. Blue Rubber Bleb Nevus (BRBN) Syndrome Is Caused by Somatic TEK (TIE2) Mutations. J Invest Dermatol. 2017;137(1):207–16. 27519652 10.1016/j.jid.2016.07.034

[37] Michel ME, Konczyk DJ, Yeung KS, Murillo R, Vivero MP, Hall AM, et al. Causal somatic mutations in urine DNA from persons with the CLOVES subgroup of the PIK3CA-related overgrowth spectrum. Clin Genet. 2018;93(5):1075–80. 29231959 10.1111/cge.13195 PMC5899663

[38] Riviere JB, Mirzaa GM, O’Roak BJ, Beddaoui M, Alcantara D, Conway RL, et al. De novo germline and postzygotic mutations in AKT3, PIK3R2 and PIK3CA cause a spectrum of related megalencephaly syndromes. Nat Genet. 2012;44(8):934–40. 22729224 10.1038/ng.2331 PMC3408813

[39] Biderman Waberski M, Lindhurst M, Keppler-Noreuil KM, Sapp JC, Baker L, Gripp KW, et al. Urine cell-free DNA is a biomarker for nephroblastomatosis or Wilms tumor in PIK3CA-related overgrowth spectrum (PROS). Genet Med. 2018;20(9):1077–81. 29300373 10.1038/gim.2017.228 PMC9365240

[40] Zenner K, Jensen DM, Cook TT, Dmyterko V, Bly RA, Ganti S, et al. Cell-free DNA as a diagnostic analyte for molecular diagnosis of vascular malformations. Genet Med. 2021;23(1):123–30. 32884133 10.1038/s41436-020-00943-8 PMC7796969

[41] Palmieri M, Pinto AM, di Blasio L, Curro A, Monica V, Sarno LD, et al. A pilot study of next generation sequencing-liquid biopsy on cell-free DNA as a novel non-invasive diagnostic tool for Klippel-Trenaunay syndrome. Vascular. 2021;29(1):85–91. 32588787 10.1177/1708538120936421

[42] Ten Broek RW, Eijkelenboom A, van der Vleuten CJM, Kamping EJ, Kets M, Verhoeven BH, et al. Comprehensive molecular and clinicopathological analysis of vascular malformations: A study of 319 cases. Genes Chromosomes Cancer. 2019;58(8):541–50. 30677207 10.1002/gcc.22739 PMC6594036

[43] Keppler-Noreuil KM, Sapp JC, Lindhurst MJ, Parker VE, Blumhorst C, Darling T, et al. Clinical delineation and natural history of the PIK3CA-related overgrowth spectrum. Am J Med Genet A. 2014;164(7):1713–33. 10.1002/ajmg.a.36552 PMC4320693 24782230

[44] Brouillard P, Schlögel MJ, Homayun Sepehr N, Helaers R, Queisser A, Fastré E, et al. Non-hotspot PIK3CA mutations are more frequent in CLOVES than in common or combined lymphatic malformations. Orphanet J Rare Dis. 2021;16(1):267. 34112235 10.1186/s13023-021-01898-y PMC8194016

[45] Blesinger H, Kaulfuß S, Aung T, Schwoch S, Prantl L, Rößler J, et al. PIK3CA mutations are specifically localized to lymphatic endothelial cells of lymphatic malformations. PLoS ONE. 2018;13(7): e0200343. 29985963 10.1371/journal.pone.0200343 PMC6037383

[46] Li D, Sheppard SE, March ME, Battig MR, Surrey LF, Srinivasan AS, et al. Genomic profiling informs diagnoses and treatment in vascular anomalies. Nat Med. 2023;29(6):1530–9. 37264205 10.1038/s41591-023-02364-x PMC11184491

[47] Balan J, Koganti T, Basu S, Dina MA, Artymiuk CJ, Barr Fritcher EG, et al. MICon Contamination Detection Workflow for Next-Generation Sequencing Laboratories Using Microhaplotype Loci and Supervised Learning. J Mol Diagn. 2023;25(8):602–10. 37236547 10.1016/j.jmoldx.2023.05.001

[48] Fiévet A, Bernard V, Tenreiro H, Dehainault C, Girard E, Deshaies V, et al. ART-DeCo: easy tool for detection and characterization of cross-contamination of DNA samples in diagnostic next-generation sequencing analysis. Eur J Hum Genet. 2019;27(5):792–800. 30683922 10.1038/s41431-018-0317-x PMC6461872

[49] Singh RR. Next-Generation Sequencing in High-Sensitive Detection of Mutations in Tumors: Challenges, Advances, and Applications. J Mol Diagn. 2020;22(8):994–1007. 32480002 10.1016/j.jmoldx.2020.04.213

[50] Steiert TA, Parra G, Gut M, Arnold N, Trotta JR, Tonda R, et al. A critical spotlight on the paradigms of FFPE-DNA sequencing. Nucleic Acids Res. 2023;51(14):7143–62. 37351572 10.1093/nar/gkad519 PMC10415133

[51] Kennedy SR, Schmitt MW, Fox EJ, Kohrn BF, Salk JJ, Ahn EH, et al. Detecting ultralow-frequency mutations by Duplex Sequencing. Nat Protoc. 2014;9(11):2586–606. 25299156 10.1038/nprot.2014.170 PMC4271547

[52] Douzgou S, Rawson M, Baselga E, Danielpour M, Faivre L, Kashanian A, et al. A standard of care for individuals with PIK3CA-related disorders: An international expert consensus statement. Clin Genet. 2022;101(1):32–47. 34240408 10.1111/cge.14027 PMC8664971

[53] Integrative genomics viewer (IGV). Available from: https://igv.org/.

[54] Schönewolf-Greulich B, Karstensen HG, Hjortshøj TD, Jørgensen FS, Harder KM, Frevert S, et al. Early diagnosis enabling precision medicine treatment in a young boy with PIK3R1-related overgrowth. Eur J Med Genet. 2022;65(10): 104590. 35964931 10.1016/j.ejmg.2022.104590

[55] Eijkelenboom A, van Schaik FMA, van Es RM, Ten Broek RW, Rinne T, van der Vleuten C, et al. Functional characterisation of a novel class of in-frame insertion variants of KRAS and HRAS. Sci Rep. 2019;9(1):8239. 31160609 10.1038/s41598-019-44584-7 PMC6547725

[56] Leichsenring J, Horak P, Kreutzfeldt S, Heining C, Christopoulos P, Volckmar AL, et al. Variant classification in precision oncology. Int J Cancer. 2019;145(11):2996–3010. 31008532 10.1002/ijc.32358

[57] Mateo J, Chakravarty D, Dienstmann R, Jezdic S, Gonzalez-Perez A, Lopez-Bigas N, et al. A framework to rank genomic alterations as targets for cancer precision medicine: the ESMO Scale for Clinical Actionability of molecular Targets (ESCAT). Ann Oncol. 2018;29(9):1895–902. 30137196 10.1093/annonc/mdy263 PMC6158764

[58] Froyen G, Le Mercier M, Lierman E, Vandepoele K, Nollet F, Boone E, et al. Standardization of Somatic Variant Classifications in Solid and Haematological Tumours by a Two-Level Approach of Biological and Clinical Classes: An Initiative of the Belgian ComPerMed Expert Panel. Cancers (Basel). 2019;11(12). 10.3390/cancers11122030 PMC6966529 31888289

[59] Lai A, Soucy A, El Achkar CM, Barkovich AJ, Cao Y, DiStefano M, et al. The ClinGen Brain Malformation Variant Curation Expert Panel: Rules for somatic variants in AKT3, MTOR, PIK3CA, and PIK3R2. Genet Med. 2022;24(11):2240–8. 35997716 10.1016/j.gim.2022.07.020 PMC9883838

