# Correction to: Assessment of gene–disease associations and recommendations for genetic testing for somatic variants in vascular anomalies by VASCERN-VASCA

**DOI:** 10.1186/s13023-024-03296-6

**Published:** 2024-10-07

**Authors:** Nicole Revencu, Astrid Eijkelenboom, Claire Bracquemart, Pia Alhopuro, Judith Armstrong, Eulalia Baselga, Claudia Cesario, Maria Lisa Dentici, Melanie Eyries, Sofia Frisk, Helena Gásdal Karstensen, Nagore Gene‑Olaciregui, Sirpa Kivirikko, Cinzia Lavarino, Inger‑Lise Mero, Rodolphe Michiels, Elisa Pisaneschi, Bitten Schönewolf‑Greulich, Ilse Wieland, Martin Zenker, Miikka Vikkula

**Affiliations:** 1 Center for Human Genetics, Cliniques universitaires Saint-Luc, University of Louvain, VASCERN VASCA European Reference Centre, Brussels, Belgium; 2 Department of Pathology, Radboud University Medical Center, VASCERN VASCA European Reference Centre, PO Box 9101, Nijmegen, 6500 HB the Netherlands; 3 Normandie Univ, UNICAEN, Service de Génétique, CHU Caen Normandie, VASCERN VASCA European Reference Centre, Caen, BIOTARGEN EA 7450, 14000 France; 4 HUS Diagnostic Center, Laboratory of Genetics, University of Helsinki and Helsinki University Hospital, VASCERN VASCA European Reference Centre, Helsinki, Finland; 5 Institut de Recerca Sant Joan de Déu, Esplugues de Llobregat, CIBER‑ER (Biomedical Network Research Center for Rare Diseases), Instituto de Salud Carlos III (ISCIII), Madrid, and Genomic Unit, Molecular and Genetic Medicine Section, Hospital Sant Joan de Déu, VASCERN VASCA European Reference Centre, Barcelona, Spain; 6 Department of Dermatology, Hospital Sant Joan de Deu, VASCERN VASCA European Reference Centre, Barcelona, Spain; 7 Laboratory of Medical Genetics, Translational Cytogenomics Research Unit, Bambino Gesù Children Hospital and Research Institute, IRCCS, VASCERN VASCA European Reference Centre, Rome, Italy; 8 Medical Genetics Unit, Bambino Gesù Children’s Hospital, IRCCS, VASCERN VASCA European Reference Centre, Rome, 00165 Italy; 9 Sorbonne Université, Département de Génétique, Assistance Publique-Hôpitaux de Paris, Hôpital Pitié- Salpêtrière, VASCERN VASCA European Reference Centre, Paris, France; 10 Department of Molecular Medicine and Surgery, Karolinska Institutet, Department of Clinical Genetics, Karolinska University Hospital, VASCERN VASCA European Reference Centre, Stockholm, Sweden; 11 Department of Genetics, Center of Diagnostics, Copenhagen University Hospital - Rigshospitalet, VASCERN VASCA European Reference Centre, Copenhagen, Denmark; 12 Laboratory of Molecular Oncology, Pediatric Cancer Center Barcelona, Hospital Sant Joan de Déu, VASCERN VASCA European Reference Centre, Barcelona, Spain; 13 Department of Clinical Genetics, HUS Diagnostic Center, University of Helsinki and Helsinki University Hospital, VASCERN VASCA European Reference Centre, Helsinki, Finland; 14 Department of Medical Genetics, Oslo University Hospital, VASCERN VASCA European Reference Centre, Oslo, Norway; 15 Laboratory of Medical Genetics, Translational Cytogenomics Research Unit, Bambino Gesù Children Hospital and Research Institute, IRCCS, VASCERN VASCA European Reference Centre, Rome, Italy; 16 Institute of Human Genetics, University Hospital Otto-Von-Guericke-University, Magdeburg, Germany; 17 Center for Vascular Anomalies, Cliniques Universitaires Saint-Luc, Brussels, Belgium; 18 Human Molecular Genetics, de Duve Institute, University of Louvain, VASCERN VASCA European Reference Centre, Brussels, Belgium; 19 WELBIO Department, WEL Research Institute, Avenue Pasteur, 6, Wavre, 1300 Belgium

**Correction to: Revencu et al. Orphanet Journal of Rare Diseases**

10.1186/s13023-024-03196-9

Following publication of the original article [1], we have been notified that there was a mistake in supplementary and additional materials’ references.

It should be as per below:

Supplementary file 1 contains Table S1

Supplementary file 2 contains Additional file 1: Curations of each gene–disease asso­ciation

Supplementary file 3 contains Table S2

Supplementary file 4 contains Table S3

## References

[1] Revencu et al. (2024) Assessment of gene–disease associations and recommendations for genetic testing for somatic variants in vascular anomalies by VASCERN-VASCA (2024). 19:213 10.1186/s13023-024-03196-9 10.1186/s13023-024-03196-9 PMC11110196 38778413

