# Supplementary material for: Assessment of gene–disease associations and recommendations for genetic testing for somatic variants in vascular anomalies by VASCERN-VASCA
Source: Orphanet J Rare Dis. 2024 May 22;19:213. doi: 10.1186/s13023-024-03196-9 (PMC11110196; doi:10.1186/s13023-024-03196-9)
Supplement: Supplementary file 1 — Supplementary Material 1. [file 13023_2024_3196_MOESM1_ESM.pdf]

Table S1 : Gene-Disease Validity Curation for Mosaic Variants

Gene:

Phenotype (ISSVA):

also applies for ISSVA phenotypes:

1. Genetic evidence

1.1. Case-level data

1.1.1. Variant evidence

Reference	Variant		Reported as	Mosaic Variant	Upgrade		Score given
**PMID, case ID; internal data**	**cDNA**	**protein**		**Basic Score: 0.5**	**Pathogenicity: 0-1.5**	**Clustering: 0-1**	
For internal data, refer to internal case-level data form			(specify phenotype designation according to reference, if applicable)		Prediction weak: +0; Prediction strong: +0.5; Functional effect: +0.5-1; Known LPATH: +1; Known PATH: +1.5	Second observation with same or other variant at same codon: +1; Neighbouring codons (+/-2): +0.5	
				0			0
				0			0
				0			0
				0			0
				0			0
				0			0
				0			0
				0			0
				0			0
				0			0
				0			0
				0			0
	Total score (maximum score for genetic case level evidence: 12)						0

2. Experimental evidence

Select, if curation of experimental evidence was not or only partially done

Score only experimental evidence that applies for the given type of vascular anomaly (experimental evidence often applies for many/all types of vascular anomalies, unless the respective experiment is specific for one type of vascular anomaly)

Category	Evidence Type	Default Score (Range)	Description	Reference (PMID)	Score given
2.1. Function	Biochemical function	0.5 (0-2)			
	Protein interaction	0.5 (0-2)			
	Expression	0.5 (0-2)			
2.2. Functional Alteration	Patient cells	1 (0-2)			
	Non-patient cells	0.5 (0-1)			
2.3. Models	Non-human model organism	2 (0-4)			
	Cell culture model	1 (0-2)			
2.4. Rescue	Rescue in human	2 (0-4)			
	Rescue in non-human model organism	2 (0-4)			
	Rescue in cell culture model	1 (0-2)			
	Rescue in patient cells	1 (0-2)			
Total score 2.1. (maximum score for category: 2)					0
Total score 2.2. (maximum score for category: 2)					0
Total score 2.3. & 2.4. (maximum combined score for categories: 4)					0

3. Clinical Validity Summary Matrix

		Summary
	Genetic evidence (0 to 12)	0
	Experimental evidence (0 to 6)	0
	Total points (sum of genetic & experimental evidence)	0
	Replication over time: >2 publ. w/ convincing evidence over time (>3 yrs) (Y/N)	
	Indicate PMID (replication 1)	
	Indicate PMID (replication 2)	
	Indicate PMID (replication 3)	
	**Calculated classification**	**NO EVIDENCE**
	Valid contradictory evidence (Y/N))	
	if Y, list PMIDs for contradictory evidence	
	if Y, describe contradictory evidence (if applicable)	
	**Curator classification**	**NO EVIDENCE**
	**Final classification (to be entered manually after panel discussion)**	

Commentary:

Please enter comments regarding this gene–disease association here if applicable. This is particularly important if the curation is considered to be applicable also for other ISSVA phenotypes in addition to one that is primarily the subject of curation in part 1 ("Genetic evidence").

Select, if curation of experimental evidence was not or only partially done
No scorable experimental evidence exists at the time of curation
Experimental evidence exists but was not curated because of sufficient scores for genetic evidence
Experimental evidence was only partially curated because of sufficient scores for genetic evidence
