# Supplementary material for: Assessment of gene–disease associations and recommendations for genetic testing for somatic variants in vascular anomalies by VASCERN-VASCA
Source: Orphanet J Rare Dis. 2024 May 22;19:213. doi: 10.1186/s13023-024-03196-9 (PMC11110196; doi:10.1186/s13023-024-03196-9)
Supplement: Supplementary file 3 — Supplementary Material 3. [file 13023_2024_3196_MOESM3_ESM.docx]

Table S2: Variants, exons and regions of interest for vascular malformations caused by somatic events (extended)

| **Gene Name** | **Transcript Ref** | **Minimal required regions** | | **PMID References** | **Optional regions** | | **PMID reference/ databases** |
| --- | --- | --- | --- | --- | --- | --- | --- |
|  |  | **Exons** | **amino acid residues of interest** |  | **Exons** | **amino acid residues of interest** |  |
| *AKT1* | NM_001382430.1 | 3 | E17 | 21793738, 33030203, 30677207 | 3  4 | L52  Q79 | Cosmic |
| *AKT3* | NM_5465.7 | 3 | E17 | 34237354, 28969385, 33309330, 25722288 | None |  |  |
| *ARAF* | NM_001654.5 | 7 | S214 | 3126328 | 9 | R255Gfs*37 | Cosmic |
| *BRAF* | NM_004333.4 | 15 | V600 | 29316280, 30544177, 29461977, 31891627, 35238086, 30544177, 35238086 | 6  11  14  15  16 | T244 to F247, Q257 G466, G469  N581  N581, D594, L597, K601  Q636 | 29493581  Cosmic  CancerHotspots |
| *CBL* | NM_005188.4 | 15 | Y774 | 32894644 | 2  7  8  9  16 | Y102  D359_K362del  Multiple hotspots, splice  C404, C416, R420, splice  A877 | ClinVar  LOVD  Cosmic |
| *GJA4* | NM_002060.3 | 2 | G41 | 33912852, 35902510, 35642047  ClinVar miner | None |  |  |
| *GNA11* | NM_002067.5 | 4  5 | R183  Q209 | 31726051, 28120216, 26778290  34040639, 26778290, 30677207, 31838126 | None |  |  |
| *GNA14* | NM_004297.4 | 4 | Q205 | 29574926, 27476652 | None |  |  |
| *GNAQ* | NM_002072.5 | 4  5 | R183  Q209 | 23656586, 25188413, 25374402, 28084343, 29174369, 30677207, 30870248, 31336681, 31532024, 31726051, 31838126, 35635655 | 2 | M59, T96 | Cosmic |
| *GNB2* | NM_005273.4 | 5 | K78 | 34124757 | None |  |  |
| *HRAS* | NM_005343.4 | 2  3 | G12, G13  T58 to M72 (small in frame insertion/ duplication) | 32043616, 34109654, 31160609, 31637524 | 3  5 | Q61  F156 | Cosmic  CancerHotspots  Clinvar VCV001198752.2 |
| *IDH1* | NM_005896.4 | 4 | R132 | 22057234, 23485734, 30677207 | None |  |  |
| *IDH2* | NM_002168.4 | 4 | R172 | 22057234, 23485734 | 4 | R140 | Cosmic  CancerHotspots |
| *KRAS* | NM_004985.5 | 2  3  4 | K5, G12, G13, Q22  Q61, Y64, T74 (small in frame insertion/ duplication)  A146 | 29298116, 31486960, 34114335, 32799314, 34056834, 34190333, 32591603, 32043616, 31026472, 30677207, 34607843, 34703427, 30544177, 35238086, 29461977, 31160609, 32859736, 34214981 | 2  3  4  5 | V14, L19  T58, A59, G60  K117  D153, F156 | 17056636, 20949621  Cosmic  CancerHotspots |
| *MAP2K1* | NM_002755.4 | 2  3 | F53_Q58delinsL, Q56, K57, K57_G61del, Q58_E62del  P105_I107delinsL, C121, P124, G128 | 28190454, 29461977, 35238086, 31486960, 30382944, 34726260, 30566190, 33747785 | 2  3  5  6  7  8 | L42, F53, D67, K59del  E102, R108, I111, H119, N122, Y130, M146  L177  E203  P264, P294  G301, S331 | 25351745  Cosmic  CancerHotspots  LOVD  ClinVar Miner |
| *MAP3K3* | NM_002401.5 | 13 | I441 | 25728774, 33899768, 33729480, 33891857  ClinVar, LOVD | 15 | Y544 | 32380920 |
| *NRAS* | NM_002524.5 | 3  4 | Q61  A146 | 32043616, 30677207, 31887709, 30542204, 29397482, 25695684 | 2  3 | G12, G13, I24, P34  T50, T58, G60 | Cosmic  CancerHotspots  LOVD  ClinVar Miner |
| *PIK3CA* | NM_006218.4 | Full gene  10  21 | 3 hotspots represent around 85% of vascular malformation variants:  E542, E545  H1047 | 34112235 | Full gene |  |  |
| *PIK3CD* | NM_005026.5 | 16 | L666 | 33964933 | 13  24 | S520  E1021 | Cosmic |
| *PIK3R1* | NM_181523.3 | 11  13  14 | Y452_Q455delins, D464_Y467del  N564, K567, Q579_Y580del  W583, splice | 34040190, 29174369, 35964931  ClinVar Miner | 9  10  13 | R348  G376  N564, K567, splice | Cosmic  CancerHotspots |
| *PTPN11* | NM_002834.5 | 3  12  13 | E76  V428, A461  T507 | doi.org/10.1016/j.gimo.2023.100562  36566878, 35778969 | 1_13 | Multiple hotspots | ClinVar  LOVD  Cosmic  CancerHotspots  29493581 |
| *TEK* | NM_00459.5 | 17  22  23 | Y897, L914, R915, S917, R918, V919, A925  R1099X  T1105, T1106, Y1108X, E1109*, E1109Lfs*5, G1115X | 27519652, 30677207, 19079259, 21962923, 23801934, 19888299, 26319232, 27030595, 34649969, 27519652, 34850385 27519652, 30677207, 19079259, 21962923, 23801934, 19888299, 26319232, 27030595, 34649969, 27519652, 34850385  ClinVar Miner, LOVD | 15  17  18  23 | R849W  L920  F960  K1100, T1105Mfs*5, T1106Hfs*4, T1112Cfs*2, Y1113Rfs*3, Y1113* | 19888299, 8980225, 10369874 |

This table describes regions of interest (genes, exons, amino acids) that should be studied for somatic exploration of vascular anomalies. The regions of interest are divided into two groups: “Minimal required regions” and “Optional regions”. The first one represents what is well described and implicated in the pathogenicity of somatic vascular anomalies, and the second one represents hotspot variants reported in cancer, or germline hotspots in RASopathy genes and *TEK*, with insufficient evidence of pathogenicity in non-hereditary vascular anomalies. Reference transcripts are from ensembl.org. References are indicated for each gene as PMID or doi from literature. Databases have also been used: LOVD, Clinvar Miner, Cosmic (v96) and CancerHotspots (v2).
