# Supplementary material for: Assessment of gene–disease associations and recommendations for genetic testing for somatic variants in vascular anomalies by VASCERN-VASCA
Source: Orphanet J Rare Dis. 2024 May 22;19:213. doi: 10.1186/s13023-024-03196-9 (PMC11110196; doi:10.1186/s13023-024-03196-9)
Supplement: Supplementary file 4 — Supplementary Material 4. [file 13023_2024_3196_MOESM4_ESM.docx]

Table S3 : Laboratory approaches and techniques used by the 11 participating centres

| Laboratory | | **1** | **2** | **3** | **4** | **5** | **6** | **7** | **8** | **9** | **10** | **11** |
| --- | --- | --- | --- | --- | --- | --- | --- | --- | --- | --- | --- | --- |
| **Material** | Histological examination | Variable  (in pathology lab) | Variable  (in pathology lab) | Variable  (in pathology lab) | Variable  (in pathology lab) | Variable  (in pathology lab) | Yes | Yes | Variable  (in pathology lab) | variable  (in pathology lab) | Variable  (in pathology lab) | Variable  (in pathology lab) |
|  | FFPE^[[1]](#footnote-2)^ | Yes | Yes, but uncommon | Yes | Yes | Yes | Yes | No | Yes | Rarely | Yes, but uncommon | Yes, but uncommon |
|  | (Micro)dissection | No | Yes | No | No | No | Yes | No | No | No | No | No |
|  | Fresh tissue | Yes | Yes | Yes | Yes | Yes | Yes, but uncommon | No | Yes | Yes | Yes | Yes |
|  | Frozen tissue | Yes | Yes, but uncommon | Yes | Yes | Yes | Yes, but uncommon | Yes | Yes | Yes, but uncommon | Yes | No |
| **DNA isolation and input** | Isolation method (manual / automated) | Manual | Automated | Automated | Manual and automated | Automated | Manual | Manual | Automated | Automated | Automated | Automated |
|  | FFPE  kit (company) | QIAmp FFPE Tissue Kit (Qiagen) | MAGCORE® Genomic DNA FFPE One-Step Kit (Atrida) | Maxwell® RSC DNA FFPE Kit  (Promega Corporation ©) | Maxwell®  (Promega Corporation ©) | QIAamp FFPE Tissue Kit (Qiagen©) | Custom, Lysis with Proteinase K,  Ethanol Precipitation | NA | Maxwell® RSC DNA FFPE Kit  (Promega Corporation ©) | NA | NA | Maxwell 16 FPPE Tissue LEV DNA Purification Kit (Promega) |
|  | Fresh/frozen tissue  kit (company) | QIAmp DNA Kit  (Qiagen) | QIAsymphony DNA mini kit (Qiagen) | Maxwell® RSC Blood DNA Kit  (Promega Corporation ©) | Phenol-Chloroform | QIAamp DNA Kit (Qiagen©) | Frozen: see FFPE,  Fresh: is usually isolated elsewhere | QIAamp DNA mini Kit (Qiagen©) | Maxwell® RSC Blood DNA Kit  (Promega Corporation ©) | NA | EZ1&2 DNA Tissue Kit  (Qiagen©) | Maxwell 16 LEV Blood DNA kit |
|  | Optimal DNA amount  (minimum DNA amount) | 200 ng  (50 ng) | 40 ng  (20 ng) | 20 ng  (6 ng) | 30 ng  (30 ng) | 100 ng  (50 ng) | 10 to 70 ng  (5 ng) | 200 ng  (200 ng) | 100 ng  (100 ng) | 100 ng  (100 ng) | 200 ng  (5 ng) | 200 ng  (200 ng) |
| **Somatic panel** | Custom/commercial  (company) | Custom  (Agilent Technologies©) | Custom  (Qiagen©) | Custom  (Thermo Fisher Scientific©) | Custom  (Thermo Fisher Scientific©) | Custom  (Agilent Technologies©) | Custom (no company, smMIP based library prep is completely custom) | Custom  (Roche Sequencing©) | Custom  (Roche Sequencing©) | Custom (Twist Bioscience) | Custom  (Agilent Technologies©) | Commercial (Agilent SureSelect v5) |
|  | Amplification or Capture | Capture | Amplification | Amplification | Amplification | Capture | Amplification | Capture | Capture | Capture | Capture | Amplification and Capture |
|  | Use UMIs (yes or no) | No | No | No | No | Yes | Yes | No | No | No | No | No |
|  | Number of genes (approximate size, kb) in library | 61  (255 kb) | 44  (37 kb) | 25  (14 kb) | 7  (23 kb) | 49  (37 kb) | 18  (10 kb) | 169  (513 kb) | 106  (361 kb) | Exome  (37 Mb) | Exome | Exome (50 Mb) |
|  | Number of genes (approximate size, kb) in virtual panel | 61  (297 kb) | 19  (12 kb) | 8  (5 kb) | 3  (7 kb) | 49  (37 kb) | 18  (10 kb) | 49  (150 kb) | 56  (146 kb) | 64  (179 kb) | 123 (combined with somatic overgrowth genes) | 69  (203.8 kb) |
| **Equipment** | Sequencing system  (company) | Miseq/MiniSeq  (Illumina®) | MiSeq  (Illumina®) | S5 Prime  (Thermo Fisher Scientific©) | S5 Prime /Proton  (Thermo Fisher Scientific©) | NextSeq 500  (Illumina©) | NovaSeq 6000  (Illumina©) | NextSeq 550  (Illumina©) | NextSeq 500  (Illumina©) | NovaSeq 6000  (Illumina©) | NovaSeq  (Illumina©) | NovaSeq6000 (Illumina®) |
|  | Paired-end or  single-end | Paired-end | Paired-end | Single-end | Single-end | Paired-end  (2 x 150 bp) | Paired-end  (2 x 150 bp) | Paired-end  (2 x 150 bp) | Paired-end | Paired-end  (2 x 100 bp) | Paired-end | Paired-end |
|  | FFPE  kit (company) | QIAmp FFPE Tissue Kit (Qiagen) | MAGCORE® Genomic DNA FFPE One-Step Kit (Atrida) | Maxwell® RSC DNA FFPE Kit  (Promega Corporation ©) | Maxwell®  (Promega Corporation ©) | QIAamp FFPE Tissue Kit (Qiagen©) | Custom, Lysis with Proteinase K,  Ethanol Precipitation | NA | Maxwell® RSC DNA FFPE Kit  (Promega Corporation ©) | NA | NA | Maxwell 16 FPPE Tissue LEV DNA Purification Kit (Promega) |
|  | Fresh/frozen tissue  kit (company) | QIAmp DNA Kit  (Qiagen) | QIAsymphony DNA mini kit (Qiagen) | Maxwell® RSC Blood DNA Kit  (Promega Corporation ©) | Phenol-Chloroform | QIAamp DNA Kit (Qiagen©) | Frozen: see FFPE,  Fresh: is usually isolated elsewhere | QIAamp DNA mini Kit (Qiagen©) | Maxwell® RSC Blood DNA Kit  (Promega Corporation ©) | NA | EZ1&2 DNA Tissue Kit  (Qiagen©) | Maxwell 16 LEV Blood DNA kit |
|  | Optimal DNA amount  (minimum DNA amount) | 200 ng  (50 ng) | 40 ng  (20 ng) | 20 ng  (6 ng) | 30 ng  (30 ng) | 100 ng  (50 ng) | 10 to 70 ng  (5 ng) | 200 ng  (200 ng) | 100 ng  (100 ng) | 100 ng  (100 ng) | 200 ng  (5 ng) | 200 ng  (200 ng) |
| **Bioinfor-matic pipelines** | Software (company) | In-house pipeline | SophiaDDM (SophiaGenetics) | Ion Torrent (Thermo Fisher Scientific©)  NextGene ®  (SoftGenetics©) | Torrent Suite Software  Ion Reporter | varvis (Limbus Medical Technologies, Rostock, Germany | Seuence Pilot (JSI Medical systems), Alamut Visual (SophiaGenetics) and in-house pipelines | DRAGEN Somatic Pipeline (Illumina) | Genodiag® | In house pipeline | In-house pipeline | In-house pipeline (based on GATK Best Practice) |
|  | Minimum coverage depth | 500 X | 500 X | 100 X | 100 X | 300 X (FFPE)  2000 X (native)  (demultiplexed UMI reads) | 250 X | 500 X | 200 X | 200 X | 400 X | 120 X^[2]^ |
|  | Minimum variant allele fraction (minimum mutant reads) | 2% (10 reads) | 2%  (10 reads) | 3%  (15 reads) | 2%  (10 reads) | 1-2%  (6 reads FFPE/ 20 reads native) | 1%  (3 unique consensus reads) | 1%  (5 reads) | 2% (10 reads) | 2%  (5 unique consensus reads) | 1% | Variants with all allele fractions predicted by the pipeline are provided ^[3]^ |
| **Through-put in routine diagnosis** | Number of runs / week | 1 | Variable | 1 | Variable | Variable | 2 | Variable | Variable | Variable | 1 | Variable |
|  | Number of samples / year | 50 | 120 | 20 | 50 | 100 | 200 | 100 | 100 | 30 | 150 | 30 |
| **Turn around time** | Minimum and maximum TAT | 15 to 20 days | 4-12 weeks | -2-6 weeks | 4 weeks | Variable | 4-11 working days | 1 week | Variable | Variable | 3 weeks – 12 weeks | Variable |

^[1]^ FFPE : formalin-fixed paraffin-embedded

^[2]^ Average coverage depth for PIK3CA is about 300× to 700×

^[3]^ All variations with an allele fraction under 10% are validated by a second method

1. [↑](#footnote-ref-2)
